# Temporal Trend of Near Miss and its Regional Variations in Brazil from 2010 to 2018

**DOI:** 10.1055/s-0040-1719144

**Published:** 2021-01-19

**Authors:** Maria Carolina Wensing Herdt, Flávio Ricardo Liberal Magajewski, Andressa Linzmeyer, Rafaela Rodolfo Tomazzoni, Nicole Pereira Domingues, Milla Pereira Domingues

**Affiliations:** 1 Universidade do Sul de Santa Catarina, Tubarão, SC, Brazil

**Keywords:** near miss, maternal mortality, hospital records, complications of pregnancy, morbidity, *near miss*, mortalidade materna, registros hospitalares, complicações da gravidez, morbidade

## Abstract

Cases of maternal near miss are those in which women survive severe maternal complications during pregnancy or the puerperium. This ecological study aimed to identify the temporal trend of near-miss cases in different regions of Brazil between 2010 and 2018, using data from the Hospital Information System (HIS) of the Unified Brazilian Health System (SUS, in the Portuguese acronym). Hospital admission records of women between 10 and 49 years old with diagnosis included in the 10 ^th^ Revision of the International Statistical Classification of Diseases and Related Health Problems (ICD-10) and codes indicating near-miss events were selected. From 20,891,040 admissions due to obstetric causes, 766,249 (3.66%) near-miss cases were identified, and 31,475 women needed admission to the intensive care unit (ICU). The cases were found to be more predominant in black women over 35 years old from the North and Northeast regions. There was a trend of increase in near-miss rates of ~ 13.5% a year during the period of the study. The trend presented a different behavior depending on the level of development of the region studied. The main causes of near miss were preeclampsia (47%), hemorrhage (24%), and sepsis (18%).

## Resumo

Casos de *near miss* materna são aqueles em que as mulheres sobrevivem a graves complicações maternas durante a gravidez ou o puerpério. Este estudo ecológico teve como objetivo identificar a tendência temporal de casos de *near miss* em diferentes regiões do Brasil entre 2010 e 2018, utilizando dados do Sistema de Informações Hospitalares (SIH) do Sistema Único de Saúde (SUS). Foram selecionados registros de internação de mulheres entre 10 e 49 anos com diagnóstico incluído na 10ª revisão da Classificação Internacional de Doenças e Problemas Relacionados à Saúde (CID-10) e códigos indicando eventos de *near miss* . Das 20.891.040 internações por causas obstétricas, 766.249 (3,66%) casos de *near miss* foram identificados, e 31.475 mulheres necessitaram de internação na unidade de terapia intensive (UTI). Constatou-se que os casos são mais predominantes em mulheres negras com mais de 35 anos da região Norte e Nordeste. Houve uma tendência de aumento nas taxas de *near miss* de aproximadamente 13,5% ao ano durante o período do estudo. A tendência apresentou um comportamento diferente, dependendo do nível de desenvolvimento da região estudada. As principais causas de *near miss* foram pré-eclâmpsia (47%), hemorragia (24%), e sepse (18%).

## Introduction

The health of woman and child is a priority in the modern world, and losses during the pregnancy-puerperium cycle and childhood are considered unacceptable to families and society. 1 Rosendo and Roncalli 2 demonstrated that the reduction of the rates of maternal and perinatal morbidity and mortality depends on investments and the restructuring of the assistance provided to pregnant women and newborns to improve its quality, which includes training and qualification of doctors and health professionals for promotion of safer maternity. To achieve this, they must be able to manage pregnancy, childbirth, and risky situations or complications in women and/or newborns. 3

It is estimated that ~ 273,000 maternal deaths occurred in the world in 2011. However, reduction of the maternal mortality rate (MMR) has been slow, ~ 2.3% a year, since 1990. In Brazil, between 2000 and 2014, the average maternal mortality rate was 55.7 deaths/100,000 live births. Despite the good performance as a nation, it is important to take a closer look at mortality rates in the macro-regions of the country, which presented considerable disparity. From every 100,000 live births, 78.6 mothers died in the North region in 2014. The Northeast presented the second-highest maternal mortality rate (71.3 deaths/100,000 live births), followed by the Southwest (54.6 deaths/100,000 live births), Central-West (54.3 deaths/100,000 live births), and South (37.6 deaths/100,000 live births). 4

Most pregnancies evolve in a physiological and healthy way, and end in uneventful labor, but among the spectrum of healthy pregnancy and maternal death, we can identify several harmful conditions for women. 5 The Maternal Morbidity Working Group of the World Health Organization (WHO), when analyzing the epidemiology of the pregnancy-obstetric-puerperal cycle, established and validated the concept of maternal *near miss* (near maternal death) or Severe Acute Maternal Morbidity (SAMM), which are situations in which certain women almost died from complications that occurred during pregnancy, childbirth or the puerperium, but somehow survived. 6 In practical terms, a pregnant woman is considered a case of near miss when she faces serious life-threatening conditions similar to those that lead to death, but survives.

To standardize these criteria, the WHO developed a classification based on three axes of severe maternal morbidity: clinical, laboratory and management markers. In addition to this classification, there are two more widely used classifications, one elaborated by Mantel et al. 7 and Waterstone et al., 8 both being based on different approaches, with different specificities and sensitivities. The classification adopted by the WHO makes it possible to identify the most serious cases, with a higher risk of death; however, the Waterstone criteria and the Mantel criteria, by using clinical disorders or identifiable organ dysfunctions, expand the possibility of detecting the cases. 7 8

Taking into account that near miss cases occur more frequently than maternal deaths, their study allows a broader identification of the risk factors most associated with the causes of maternal mortality. 9 The identification of these cases is increasingly recognized as a useful strategy for assessing the quality of obstetric care. In other words, maternal near miss is a sensitive and relevant indicator related to women's health care, and it seems to be associated with the level of human and social development in different societies. 10

The clarification of the temporal trend of maternal near miss, which is the main age range affected and its risk factors, contributes to the expansion of knowledge on a subject that is not as much discussed, and can serve as a tool for monitoring the network and add to the endorsement of public policies that protect women from maternal complications and, consequently, reduce the mortality and morbidity rates of this group.

The question that guided this research was: which were the temporal trends of maternal near miss and its regional variations in Brazil from 2010 to 2018.

## Methods

This was an observational ecological study that analyzed temporal series of data from the Hospital Information System of the Unified Brazilian Health System (HIS/SUS, in the Portuguese acronym). Records of women between 10 and 49 years old from different regions of Brazil, admitted between 2010 and 2018, were considered. The selection was done according to the fields: main diagnosis, secondary diagnosis, macro-region, race, and admission to the ICU.

The database was composed following the algorithm presented in Table 1 , using the tabulation software, Tabwin. First, all hospital records of women living in Brazil, admitted between 2010 and 2018, were selected, totaling 59,911,177 admissions. Then, filters of age (10 to 49 years old) and main diagnosis included in Chapter XV - *Pregnancy, childbirth, and the puerperium* - of the 10 ^th^ Revision of the International Statistical Classification of Diseases and Related Health Problems (ICD-10) 11 were applied, resulting in 20,891,040 women admitted as the population of the study.

**Table 1:** Risk rates (x100 deliveries) of admissions due to a near-miss event by macroregion and year of occurrence

Year\Region	North	Northeast	Southeast	South	Central-West	Total
2010	6.40	5.65	5.31	4.78	5.57	5.52
2011	6.83	5.73	5.28	4.69	4.98	5.52
2012	6.87	5.90	5.38	4.67	4.34	5.55
2013	6.95	5.91	5.33	4.83	4.66	5.58
2014	6.94	6.12	5.16	4.61	4.13	5.52
2015	6.17	5.99	5.10	4.66	4.57	5.39
2016	6.67	6.91	5.54	5.79	5.12	6.11
2017	7.70	7.92	6.18	6.42	5.57	6.88
2018	8.17	7.89	6.42	6.61	6.08	7.11
*Mean*	*6.95*	*6.41*	*5.52*	*5.18*	*4.99*	*5.89*
*Spearman*	*0.5*	*0.97*	*0.55*	*0.53*	*0.34*	*0.64*
*Beta*	*0.63*	*0.89*	*0.71*	*0.82*	*0.37*	*0.80*
*p-value*	*0.07*	*0.00* *	*0.03* *	*0.01* *	*0.32*	*0.06*

Spearman = Spearman coefficient of correlation; Beta = mean annual variation (near-miss cases/100 deliveries/year); *p* -value (ANOVA).

* = *p* < 0.05.

To select the records of admissions due to SAMM - near miss - the ICD-10 codes corresponding to the near-miss diagnosis were used according to the criteria and definitions established by Mantel et al. 7 and Waterstone et al., 8 as seen in Chart 1 . 7 8 Mantel's criteria include conditions that are typical of organic dysfunctions in organs and human body systems as long they are related to pregnancy, childbirth, and the puerperium, whereas Waterstone's criteria include clinical diagnoses of the most frequent pathological conditions of pregnancy, childbirth, and puerperium, such as severe preeclampsia, hemorrhage, sepsis, and uterine rupture. 7 8

**Chart 1:** Near miss classification

**Mantel's criteria**	
A.1 Organ dysfunction	
Criteria/definitions	Generic categorization of diagnoses [ICD-10 Codes]
	Pulmonary edema [J81]
1. Cardiac dysfunction	Cardiomyopathy; congestive heart failure
1.1 Pulmonary edema	[I11.0; I42.0; I42.1; I42.8; I42.9; I43.8; I46; I46.0;
1.2 Cardiac Arrest	I46.9; I50.0; I50.1; I50.9; O75.4; O90.3; R57.0]
3. Immunological dysfunction	Infection; sepsis; genital tract and pelvic infection complicating abortion
3.1 Admission to the ICU for sepsis	
	Peritonitis; salpingitis [A02.1; A22.7; A26.7; A32.7;
3.2 Emergency hysterectomy for sepsis	A40; A40.0; A40.1; A40.2; A40.3; A40.8; A40.9; A41;
	A41.0; A41.1; A41.2;
	A41.3; A41.4; A41.5; A41.8; A41.9; A42.7; A54.8;
	B37.7; K35.0;
	K35.9; K65.0; K65.8; K65.9; M86.9; N70.0; N70.9;
	N71.0; N73.3;
	N73.5; O03.0; O03.5; O04.0; O04.5; O05.0; O05.5;
	O06.0;
	O06.5; O07.0; O07.5; O08.0; O08.2; O08.3; O41.1;
	O75.3; O85; O86; O86.0; O86.8; O88.3; T80.2]
4. Respiratory dysfunction	
4.1 Intubation and ventilation for more than 60 minutes except for general anesthesia	Respiratory failure; respiratory arrest; embolism
4.2 Peripheral O2 saturation < 90% for more than 60 minutes	Embolism complicating abortion [I26.9; J80; J96; J96.0;
4.3 Ratio Pa O2/ FiO2 ≤ 3 Ratio Pa O2/ FiO2 ≤ 300 mm Hg	J96.9; O03.7; O04.7; O05.2; O06.2; O06.7; O88.1;
	R09.2]
5. Renal dysfunction 5.1 Oliguria, defined as diurese < 400 ml/24 hour	Renal failure following abortion [O08.4; R34]
5.2 Acute urea deterioration to 15 mmol/l or creatinine > 400 mmol/l	Acute kidney failure [E72.2; I12.0; I13.1; I13.2;
	N17; N17.0; N17.1; N17.2; N17.8; N17.9; N18.0;
	O08.4; O90.4]
**Mantel's criteria**	
A.1 Organ dysfunction	
Criteria/definitions	Generic categorization of diagnoses [ICD-10 Codes]
6. Liver dysfunction 6.1 Jaundice during preeclampsia	Liver dysfunctions; viral hepatitis complicating pregnancy, childbirth and the puerperium [K72; K72.0; K72.9; O26.6; O98.4]
7. Metabolic dysfunction	Diabetes mellitus with coma or ketoacidosis [E10.0;
7.1 Diabetic Ketoacidosis	E10.1; E11.0; E11.1; E12.0; E12.1; E13.0; E13.1;
	E14.0; E14.1]
7.2 Thyrotoxic crisis	Thyrotoxicosis; metabolic disorder following abortion [E05; E05.0; E05.1; E05.2; E05.3; E05.4; E05.5; E05.8; E05.9; E06.0; E07; E07.8; E07.9; O08.5]
8. Coagulation dysfunction 8.1 Acute thrombocytopenia requiring transfusion of platelets	Disseminated intravascular coagulation; coagulation deficiencies [D65; D68; D68.9; D69.4; D69.5; D69.6; D82.0; O45.0; O72.3]
9.Sub-arachnoid or intracerebral hemorrhage	Intracerebral hemorrhage; stroke; vertebral venous thrombosis during pregnancy
	[G93.6; I60; I60.0; I60.1; I60.2;
	I60.3; I60.4; I60.5; I60.6; I60.7; I60.9; I61; I61.0; I61.1;
	I61.2; I61.3; I61.4; I61.5; I61.6; I61.8; I61.9; I64; I69.1;
	O22.5]
**Waterstone's criteria**	
Criteria/codes	Generic categorization of diagnoses [ICD-10 Codes]
1. Severe preeclampsia	Moderate, severe or unspecified pre-eclampsia; pre-existing hypertension with superimposed proteinuria [O11; O14.0; O14.1; O14.9]
2. Eclampsia	Eclampsia complicating pregnancy, childbirth or the puerperium [O15; O15.0; O15.1; O15.2; O15.9]
3. HELLP ^c^ syndrome	
4. Severe hemorrhage	Delayed or excessive hemorrhage complicating abortion. Placenta previa with hemorrhage. Premature separation of placenta [D62; O03.1; O03.6; O04.1; O04.6; O05.1; O05.6; O06.1; O06.6; O07.1; O07.6;O08.1; O44.1; O45.0; O45.8; O45.9; O46; O46.0; O46.8; O46.9; O67.0; O67.8; O67.9; O69.4; O72; O72.0; O72.1; O72.2]
5. Sepsis	Infection; septicemia; genital tract infection complicating abortion. Peritonitis. Salpingitis [A02.1; A22.7; A26.7; A32.7; A40; A40.0; A40.1; A40.2; A40.3; A40.8; A40.9; A41; A41.0; A41.1; A41.2; A41.3; A41.4; A41.5; A41.8; A41.9; A42.7; A54.8; B37.7; K35.0; K35.9; K65.0; K65.8; K65.9; M86.9; N70.0; N70.9; N71.0; N73.3; N73.5; O03.0; O03.5; O04.0; O04.5; O05.0; O05.5; O06.0; O06.5; O07.0; O07.5; O08.0; O08.2; O08.3; O41.1; O75.3; O85; O86; O86.0; O86.8; O88.3; T80.2]
6. Uterine rupture	Rupture of uterus before or during labor. Disruption of cesarean delivery wound [O71.0; O71.1; O90.0]
**Waterstone's criteria**	
Criteria/definitions	Generic categorization of diagnoses [ICD-10 Codes]
1. Acute abdomen	Acute abdomen [R10.0]
2. Disease caused by human immunodeficiency virus ^d^	Infection caused by the human immunodeficiency virus [B20; B20.0; B20.1; B20.4; B20.8; B20.9]

Abbreviations: ICD-10, 10th Revision of the International Statistical Classification of Diseases and Related Health Problems; ICU, Intensive care unit; HELLP syndrome, hemolysis (H), high levels of liver enzymes (EL) and low platelet count (LP).

Admission records that contained procedures regarding clinical complications of pregnancy were removed because the codes related to the complications do not discriminate their severity and could encompass any complication, even those not related to severe maternal morbidity, what would allow the same patient to be included twice (by the main diagnosis and by the procedure they were submitted to). Importantly, the admissions due to near-miss events considered were chosen based on the application of the criteria for sequential selection, eliminating the risk of duplicates.

To perform the temporal analysis of near-miss cases in Brazil, all admission records with codes included in Chapter XV of ICD-10 11 were selected, totalizing 765,824 cases. Near-miss cases with secondary diagnosis in other chapters of the ICD-10 were removed – a total of 425 cases, which is less than 0.05% of the cases considered.

For each year of the temporal trend, the rate of SAMM was calculated by dividing the number of hospital admissions due to severe maternal morbidity by the total number of deliveries during the same period, multiplied by 100. That is, the rate of SAMM = (near-miss cases/total of deliveries) *100. The denominator considered the number of deliveries included in the database according to the main diagnosis included in each inpatient hospital authorization (IHA) found at the HIS/SUS and not the number of live births, since it is not possible to distinguish between the births that are financially supported by the SUS and those that are not in the Brazilian Live Birth Information System (SINASC, in the Portuguese acronym). Only admissions supported by the SUS between 2010 and 2018 were included in this study.

The absolute and relative frequencies of admissions for near-miss events were described according to the most recent criteria. The age was stratified in 5-year intervals with the intent to estimate the frequency and near-miss rates according to different age groups in the reproductive cycle.

The average annual variation of each series, obtained by simple linear regression (Beta coefficient - β), was used to analyze the trends of severe acute maternal morbidity. The strength of the time-event correlation was obtained by calculating the Spearman's correlation coefficient. The statistical significance was calculated by the analysis of variance (ANOVA), and 95% was adopted as the significance level ( *p* < 00.5).

For being an ecological study with population aggregation analysis without research subjects and of public access, it was not necessary to subject it to registration and analysis of the Ethics Committee of Research involving Human Beings, according to Resolution no. 510/2016 of the National Health Council (CNS) (Article 1, Paragraph one of one, clauses III and V).

**Fig. 1:**
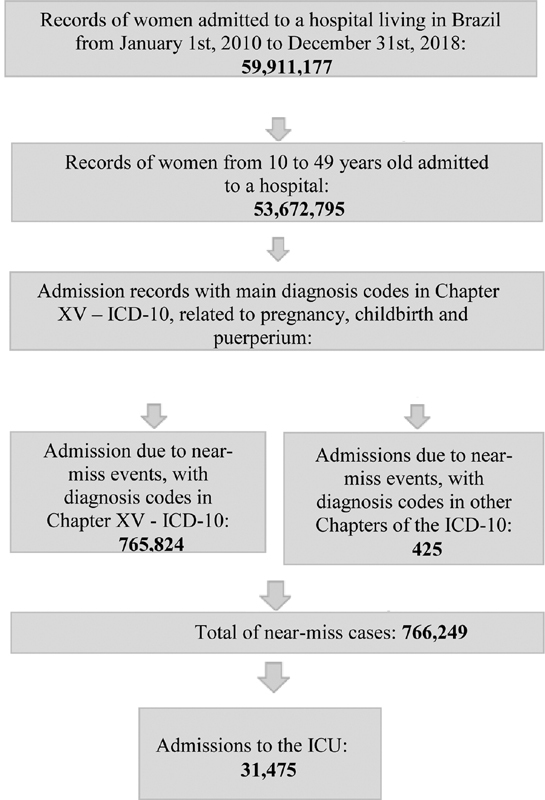
Schematic flowchart of the data collection process and selection of near-miss cases.

The collection sequence performed to meet the goals of this research can be understood more clearly in the flowchart below ( Fig. 1 ):

## Results

The retrospective research of women admitted to any hospital, anywhere in the country, due to complications related to pregnancy, childbirth and the puerperium, financially supported by the SUS, during a 9-year period (2010–2018), resulted in a total of 20,891,040 admissions. From this total, 766,249 admissions (3.66%) due to SAMM - near miss, were selected. From these cases, it was verified that 31,475 women (4.1%) needed to be admitted to the intensive care unit (ICU) ( Table 1 ).

Table 1 shows that near-miss rates presented a trend of increase in every region of Brazil. The Northeast region had the most expressive increase. The time-event correlations in all regions, except North and Central-West, represented by the Spearman correlation test, were strong and significant ( *p* < 0.05). Brazil, as a whole, presented a positive average variation of 0.80 near-miss cases per every 100 deliveries a year, which represents an increase of 13.5% a year. Women from the North region presented a risk of a near-miss event 25% higher than those from the Central-West region, which had the lowest average risk rate.

Table 2 indicates a trend of increase in all the series of rates analyzed, with statistical significance ( *p* < 0.02) from 20 years old on. The increase in risk occurred along with the increase in the maternal age, from 15 years old on, and was more prominent in the age groups 40 to 44 and 45 to 49 years old (β = 0.917 and 0.792, respectively). Moreover, patients aged 40 to 49 years old presented a chance of having a near-miss event almost 3 times higher than the age group with the lowest risk—age group 15 to 19 years old (relative risk [RR] 2.61; confidence interval [CI] 95%: 2.39–2.89; *p* < 0.001).

**Table 2:** Risk rates (x100 deliveries) of admissions due to a near-miss event by age group of the patient and year of occurrence

Year/Age group	10–14	15–19	20–29	30–39	40–44	45–49	Total
2010	6.07	4.44	4.99	7.71	11.64	18.28	5.52
2011	6.16	4.49	4.95	7.59	12.52	18.09	5.52
2012	6.04	4.47	5.02	7.53	12.22	19.28	5.55
2013	6.38	4.47	5.02	7.59	12.87	19.51	5.58
2014	5.97	4.39	4.91	7.65	12.64	19.29	5.52
2015	5.43	4.21	4.81	7.43	12.98	18.37	5.39
2016	5.70	4.56	5.42	8.65	14.24	19.52	6.11
2017	6.90	5.03	6.09	9.63	15.82	22.90	6.88
2018	6.70	5.14	6.26	9.87	16.14	22.39	7.11
*Mean*	*6.12*	*4.56*	*5.26*	*8.21*	*13.51*	*19.61*	*5.89*
*Spearman's*	*0.18*	*0.53*	*0.56*	*0.51*	*0.97*	*0.85*	*0.64*
*Beta*	*0.31*	*0.65*	*0.77*	*0.79*	*0.92*	*0.79*	*0.80*
*p-value*	*0.42*	*0.06*	*0.02*	*0.01*	*0.00*	*0.01*	*0.06*

Spearman = Spearman coefficient of correlation; Beta = mean annual variation (near-miss cases/100 deliveries/year); *p* -value (ANOVA).

With regards to the skin color of the hospitalized women, black women presented a risk of a near-miss event 19% higher than white women (RR 1.19; CI 95%: 1.06–1.33; *p* < 0.001) ( Table 2 ).

In Table 3 , considering the Waterstone's criteria, the main causes of hospitalization due to a near-miss event were preeclampsia, with a rate of 2.78 admissions per every 100 deliveries (47%), followed by severe hemorrhage (24%), sepsis (18%), eclampsia (8%), and uterine rupture (3%). 8 Except for the age group 10 to 14 years old, there was a progressive increase in complications due to a near-miss event following the increase in maternal age. Preeclampsia was the most prevalent cause of admission due to a near-miss event in every age group, followed by severe hemorrhage, predominant in the intermediate age groups and sepsis, predominant in the extreme age groups.

**Table 3:** Rates of admissions due to a near-miss event in Brazil, between 2010 and 2018, by criteria and age group, per every 100 deliveries

**Waterstone's criteria**							
Age groups							
Criteria	10 to 14	15 to 19	20 to 29	30 to 39	40 to 44	45 to 49	TOTAL
Preeclampsia	2.31	1.91	2.48	4.20	6.45	6.40	2.78
Eclampsia	0.79	0.45	0.41	0.64	0.06	0.00	0.48
Severe hemorrhage	1.40	1.04	1.30	1.96	3.66	5.89	1.43
Sepsis	1.56	1.07	097	1.29	2.25	6.09	1.10
Uterine rupture	0.06	0.04	0.05	0.08	0.13	0.36	0.05
TOTAL	6.13	4.54	5.22	8.18	13.54	19.84	5.86
**Mantel's criteria**							
Age groups							
Criteria	10 to 14	15 to 19	20 to 29	30 to 39	40 to 44	45 to 49	TOTAL
Cardiac dysfunction	0.00	0.00	0.00	0.001	0.002	0.00	0.00
Vascular dysfunction	0.00	0.00	0.00	0.00	0.00	0.00	0.00
Sepsis	1.56	1.07	0.97	1.29	2.25	6.10	1.10
Respiratory dysfunction	0.015	0.01	0.01	0.01	0.04	0.06	0.01
Abortion	0.002	0.00	0.00	0.00	0.00	0.006	0.00
Acute kidney failure	0.002	0.001	0.002	0.003	0.003	0.01	0.002
Kidney dysfunction	0.003	0.004	0.007	0.01	0.01	0.00	0.007
Diabetic Ketoacidosis	0.00	0.00	0.001	0.003	0.005	0.00	0.001
Thyrotoxicosis	0.005	0.003	0.003	0.003	0.005	0.00	0.003
Coagulation dysfunction	0.04	0.04	0.05	0.07	0.10	0.12	0.05
Cerebral dysfunction	0.002	0.001	0.002	0.003	0.003	0.01	0.002
Pulmonary dysfunction	0.01	0.01	0.01	0.015	0.02	0.04	0.01
TOTAL	1.64	1.14	1.06	1.42	2.45	6.36	1.19

The prevalence of admissions due to a near-miss event (Waterstone's criteria) according to the macro-region of occurrence highlighted important differences between them. In the Northeast region, admission for preeclampsia had an incidence 42% higher than in the Central-West region (RR 1.42; CI 95% 1.34–1.50; *p* < 0.001), whereas in the South region, eclampsia had an incidence 39% higher than in the Central-West region (RR 1.39; CI 95% 1.23–1.57; *p* < 0.001). The North region presented a relative risk of severe hemorrhage two times higher (RR 2.06; CI 95% 1.93 - 2.21; *p* < 0.001) and a 33% higher risk of sepsis when compared with the Central-West region (RR 1.33; CI 95% 1.23–1.44; *p* < 0.001). In the Southeast region, the risk of uterine rupture was 133% higher than in the Central-West region (RR 2.33; CI 95% 1.60-3.40; *p* < 0.001). The Central-West region presented the lowest risk rates for causes related to near-miss events, which is the reason why it was used as a base of comparison for the regions with higher specific risk.

Women admitted due to a maternal near miss were 31 times more likely to be admitted to the ICU (RR 31.32; CI 95%: 28.82 - 34.03, *p* < 0 0.001).

## Discussion

The present study verified a trend of increase of ~ 13.5% a year in hospital admissions due to near-miss events in Brazil during the period of the study. This trend is corroborated by a Brazilian study that analyzed the period between 2000 and 2012 and also verified an increase in the risk rates of near miss. 12

When taking into account the average rates of SAMM in Brazil during the period between 2010 and 2018, there is a risk rate of 5.89 near-miss cases per every 100 deliveries, which is higher than those of other studies that also used the HIS/SUS database. 2 3 4 5 6 7 8 9 10 11 12 13 In the population-based study of Sousa et al. in 2008, 14 they analyzed different Brazilian capitals and macro-regions and found a rate of 44.3/1,000 live births.

Nevertheless, maternal mortality in Brazil remained stable during the last few years, contrary to the positive trend in severe maternal morbidity. 14 This apparent contradiction highlights the importance of discussing near miss, as it is possible that the identification of a higher number of cases might have guaranteed more comprehensive assistance to a greater number of women in a risky obstetric situation, reducing the more severe outcomes.

The average risk rate of near miss in the Northeast region found in the present study (6.41/100 deliveries), despite using a different methodology, was higher than the estimates of SAMM presented in the study of Rosendo and Roncalli, 2 which analyzed 167 cities of the State of the Rio Grande do Norte between 2008 and 2012 and verified a near-miss rate of 36.76/1,000 obstetric admissions.

There were severe inequalities between the Brazilian macro-regions, especially in relation to human development. 15 A study on the evolution of the Human Development Index (HDI) in the Brazilian macro-regions verified that the North and Northeast regions presented the highest positive variations in every component of the HDI between 2000 and 2010, despite remaining with the lowest indexes among all Brazilian regions. 16 In a broad sense, even with the improvement of the indicators of maternal and child health care verified in several studies, socioeconomic and health-care differences are still prevalent in the North and the Northeast, which might explain the possible negative association between the highest risk rates and the lowest indexes of obstetric care verified in these regions. 9 10 11 12 13 14 15 16 17 18 19 20 Moreover, the North region presented a relative risk of hemorrhage two times higher and a 33% higher risk of of infection than the region with the lowest rates. This context suggests challenges in the access of pregnant women to health care units and specialized treatment, and fits the Three Delays Model of Thaddeus and Maine, 21 in which patients delay the search for assistance due to sociocultural reasons, are not able to access obstetric care, and when they manage to do it, they have to wait for a long time to receive treatment. In these regions, investments aimed at organizing an efficient and articulate maternal care network that offers support and qualified human resources, to provide quality care to pregnant women, are essential.

The comparative analysis of the main near-miss complications in different Brazilian macro-regions demonstrated that the Southeast region presented a 133% higher risk of uterine rupture. The fact that uterine rupture occurs more commonly in women with a c-section scar makes this complication one of the most concerning. In this sense, the increased risk can be explained by the higher prevalence of cesarean delivery in the Southeast region of the country. In a study of 2013, Eufrásio 22 verified a prevalence of 53.03% of cesarean delivery throughout Brazil, whereas in the Southeast region the prevalence was 59.32%. The high incidence of this type of delivery is concerning, as it is known that it increases the risk of neonatal and maternal morbidity and mortality and has been becoming a severe public health care problem in Brazil. 22

With regards to the South region, the RR of eclampsia was 39% higher than the region with the lowest risk, the Central-West. This puts into question the effectiveness and quality of prenatal care in the most developed regions of Brazil. Concerning prenatal care, Viellas et al., 23 studying the period between 2011 and 2012, reported a 98.7% coverage of prenatal care throughout Brazil, and nearly 100% coverage in the South region. However, several obstacles might contribute to low-quality prenatal care, such as the existence of structural barriers, unavailability of medicaments and essential exams, and problems in the provision of health-care actions involving individual attention and clinical care. 23 In relation to eclampsia, which is preceded by well-known medical signs that are easily identifiable in the prenatal examination, the question that arises is: what is reducing the effectiveness of the prenatal care offered to virtually the whole Brazilian population through the Family Health Strategy (FHS)? Concerning the age groups, the highest near-miss rates are concentrated in the population above 40 years old. This was also verified in a study by Morse that analyzed near-miss prevalence at a reference hospital in Rio de Janeiro in 2009. 24 Several data found the literature point to age as a risk factor for the occurrence of obstetric complications, a fact associated with the increase in the number of women pregnant after 40 years old. The increase in maternal age is related to the higher incidence of comorbidities, such as hypertensive disorders of pregnancy, gestational diabetes, obesity, placenta previa, and need for cesarean section, which are connected to the increase in the risk of a near-miss event. 25 A Finnish study that analyzed the period between 1997 and 2008 indicated that women in advanced age had a risk of preeclampsia 1.5 times higher than women under 40 years old. 25

Preeclampsia remained as the complication with the highest risk rates in Brazil during the period studied. Adisasmita et al. 26 also verified that 57.3% of women in Indonesia presented hypertensive syndrome as a primary determining factor. Contrarily, studies performed by Rosendo and Roncalli, 2 and Cecatti et al. 3 presented hemorrhage as the main cause of near miss. The explanation for this difference might be found in the methodology used by the studies analyzed, which were based on self-reported morbidity. Despite severe hemorrhage having a near-miss rate lower than that of preeclampsia, it suffered a trend of increase following the increase of the maternal age during the period of the study. This is a relevant fact considering that, once again, the non-recognition or delay in the identification of cases and institution of effective therapy are the only possible explanations for this reality. With resources, accurate diagnosis and assistance at the right moment, hemorrhage can be the most preventable of the maternal mortality causes. Nonetheless, barriers, such as the lack of systematization of assistance in emergencies, inadequate medical approach that underestimates blood loss, insufficient fluid resuscitation and delay in the surgical approach after errors in the clinical treatment, are quite common in obstetric centers. 27 The “Birth in Brazil” survey, performed between February 2011 and October 2012, assessed data about near miss according to the criteria of the WHO. The near-miss rate found was of 10.2/1,000 live births and 30.8 near-miss cases per every maternal death. Such findings are conservative, as cases of abortion and complications that occurred during the puerperium after the hospital discharge were not included. 28 The present study found near-miss rates almost five times higher than the aforementioned survey. The utilization of Waterstone's and Mantel's definitions widened the criteria used for the diagnosis of maternal near-miss cases, which can be considered a plausible explanation for the higher incidence found. 7 8 Regarding the result of hospitalizations for near miss, we affirm that there was a proportional tendency of increase between the risk rates of near miss and admission to the ICU in Brazil, during the studied period. In other studies, ICU admissions also showed a direct relationship with the number of maternal near miss cases, as well as an association with a worse prognosis. 2 3 4 5 6 7 8 9 10 11 12 13 14 15 16 17 18 19 20 21 22 23 24

It is important to highlight that the WHO's criteria for near miss were not used in this research due to the difficulty in correlating them with the ICD-10 diagnoses used by HIS-SUS. For the characterization of near-miss cases, the WHO proposes the use of the diagnosis of organ dysfunction, which can be revealed following clinical, laboratory and treatment criteria. 6 The choice for Waterstone's and Mantel's criteria to identify maternal near-miss cases made the correlation between the medical conditions and the ICD-10 codes, which constitute the “main diagnosis” field in the HIS-SUS, easier. 7 8 Nevertheless, the classification adopted by the WHO is more selective for severity as it identifies cases with a higher risk of death, whereas Waterstone's criteria tend to encompass a higher number of cases, even the ones that are less severe. 8 Despite being difficult to systematize the identification of near-miss cases, it is essential to understand them to plan for the assistance provided during pregnancy, childbirth, and the puerperium. Their identification reveals relevant information that health-care professionals can use to avoid maternal morbidity and mortality. Filippi et al., 29 in a study involving three countries,—Benin, Ivory Coast, and Morocco—proposed that near-miss cases should be estimated in two moments: cases identified at the arrival at hospital, as a good indicator of the obstetric care during emergencies; and cases that happened after admission, as a tool for monitoring the quality of the obstetric services. 29

It is important to emphasize that Brazil is one of the few countries that counts with a well-structured hospital information system, the HIS/SUS, which makes data of reasonable quality available for the analysis of hospital morbidity and development of preventive measures. 30 Its underutilization as diagnosis and monitoring of the improvement of the quality of obstetric care in Brazil is a reflection of the stage of Brazil's scientific and technological development. The method used in the present research proved to be suitable for the identification of near-miss cases upon analysis of the information from the HIS/UHS. These findings are corroborated by the study of Silva et al., 31 performed in the State of Paraná in 2010.

Regarding the limitations imposed on this paper resulting from the use of secondary data, we can observe that the reliability of the information collected at the SIH/SUS not only depends on the quality of the data filled in hospital records but also on the competence of professionals who register the admission diagnoses in hospitals. One should also take into account the fact that the SIH/SUS has as its main duty the directing of monetary resources to hospitals, and it is sometimes necessary to change the codes of procedures to better adjust the financial transfer. 31 Still in relation to the difficulties attributed to this work, it is important to highlight some of these characteristics of the ecological study methodology. One of the restrictive aspects concerns the databases of the morbidities researched, which may suffer the influence of the different levels of development of each region of the country and may impact the reliability of the information with qualitative errors and underreporting.

## Conclusion

The results of the present study demonstrate a trend of increase in the average risk rates of severe acute maternal morbidity in Brazil, between 2010 and 2018. The highest near-miss rates were concentrated in the North and Northeast region, and cases were more predominant among black women over 40 years old. The main near-miss causes that affected Brazilian women were preeclampsia, severe hemorrhage, sepsis, and uterine rupture, in this order. Maternal near miss stands out as a complement to the investigation of maternal mortality. Its understanding helps the elaboration of strategies for reducing maternal mortality as it allows for a quicker obtainment of information about obstetric care since women that die go through the stage of organ dysfunction earlier. Thus, near-miss cases appear as a mean that allows strategies for early diagnosis and prevention to be possible and more effective. Primary prevention policies as well as well-structured programs that guarantee equity in the access to healthcare units, diagnosis, and follow-up are essential to reverse the current scenario and reduce the burden of this morbidity in Brazilian women.
